# Comparative Assessment of Short- and Long-Term Effects of Triadimenol Fungicide on *Danio rerio* Erythrocytes Using the Micronucleus and Erythrocyte Nuclear Abnormality Assays

**DOI:** 10.3390/toxics13030199

**Published:** 2025-03-11

**Authors:** Pinar Goc Rasgele

**Affiliations:** Faculty of Agriculture, Duzce University, Duzce 81620, Türkiye; pinarrasgele@duzce.edu.tr or pinargocrasgele@gmail.com

**Keywords:** triadimenol, genotoxicity, zebrafish, genotoxicity assays, ecotoxicology

## Abstract

Triadimenol is a systemic fungicide widely used in agriculture to manage plant diseases, especially fungal infections. This study aims to evaluate the short-term (24, 48, 72 and 96 h) and long-term (10, 20, and 30 days) genotoxic effects of different concentrations of triadimenol on zebrafish (*Danio rerio*) erythrocytes using micronucleus (MN) and erythrocyte nuclear abnormal (ENA) assay. Fish were treated with 1.5, 3, and 6 mg/L concentrations of triadimenol for short and long-term periods. After the treatment period, blood was collected with heparin syringe, smears were prepared, the preparations were fixed and stained. For MN assay in short-term treatments, statistically significant MN formation was found at all concentrations of triadimenol for 24 h treatment, at the highest triadimenol concentration for 48 h, at 1.5 and 3 mg/L concentrations for 72 h, and at 3 mg/L concentrations for 96 h, compared to the negative control. In long-term treatments, significant increases in MN formation were observed at all concentrations of triadimenol for 10 and 20 days of treatment compared to the negative control. Mortality occurred at 3 and 6 mg/L concentrations in the 30-day treatment. The most frequently detected abnormalities included echinocytes and binuclear cells. For ENA assay, abnormalities such as echinocytes, binuclear cells, segmented cells, and kidney-shaped nuclei were detected in fish erythrocytes treated with different concentrations of triadimenol. All concentrations of triadimenol caused an increase in the total abnormality level in *Danio rerio* erythrocytes at all treatment times. These increases were concentration dependent for both short-term and long-term treatments. In conclusion, this study emphasized the potential genotoxic risks of triadimenol fungicide for aquatic organisms in both short-term and long-term treatments and the need for further ecotoxicological evaluation.

## 1. Introduction

Pesticides are widely used in agriculture to protect crops from various pests, fungi, and diseases. However, the potential environmental and health risks associated with pesticide use have raised significant concerns globally. Among the various classes of pesticides, fungicides are frequently applied to control fungal infections in crops. In 2020, approximately 605,986 tons of fungicides were sold worldwide, with 31% of these sales occurring in Europe [1]. Fungicides represent the second-most-sold category of agricultural pesticides globally, accounting for 23% of total pesticide sales [1]. However, their effects are often underestimated compared to those of insecticides and herbicides. Triadimenol (TDML), a member of the triazole family, is a systemic fungicide primarily used to control diseases like powdery mildew in crops. Although it is effective in crop protection, there is growing evidence suggesting that exposure to triazole-based fungicides like TDML may have adverse effects on human health and the environment [2].

TDML is a systemic fungicide extensively used in agriculture to manage plant diseases, particularly fungal infections. Despite its efficacy, the growing concern about its environmental persistence and potential toxicity to non-target organisms has made it a subject of intensive research [3]. Particularly, its genotoxicity has become an area of critical study. Genotoxic substances are capable of inducing mutations, chromosomal fragmentation, and other genetic alterations, which can lead to carcinogenesis and developmental defects. This makes the study of TDML’s genotoxic potential essential, not only for understanding its effects on human health but also for assessing its impact on biodiversity and ecosystem stability [4]. Recent studies have shown that exposure to TDML may result in genotoxic effects, such as micronucleus formation, chromosomal aberrations, and DNA strand breaks in various cell types. For instance, in vitro studies have demonstrated that TDML can cause chromosomal damage in human lymphocytes [5]. These effects suggest that long-term exposure to even low levels of TDML could pose serious risks to human health. Furthermore, its potential to induce genetic mutations in aquatic organisms raises concerns about its impact on aquatic biodiversity [6]. Given that many pesticides, including TDML, are frequently detected in water bodies, the study of their genotoxic effects in aquatic species is crucial for environmental risk assessment.

One of the most commonly used model organisms to study the effects of environmental toxins, including pesticides, on aquatic organisms is *Danio rerio*. This species is used in many different fields of study due to its transparent embryos, rapid development, and similarity of genetic pathways to humans [7,8]. Genotoxicity studies are one of these. The use of zebrafish in toxicology has allowed researchers to explore the impacts of various chemical exposures, including pesticides like TDML, on developmental processes, genetic integrity, and overall health [7]. In recent years, studies involving *D. rerio* have provided valuable insights into the genotoxic effects of fungicides, with evidence showing that these chemicals can cause significant chromosomal damage and increase the frequency of micronucleus formation, both of which are indicators of genotoxicity [9]. This highlights the relevance of using *D. rerio* as a model organism for evaluating the genotoxic effects of TDML and other pesticides, offering a more comprehensive understanding of their risks to both human health and the environment.

Erythrocyte nuclear abnormality refers to various structural alterations in the nucleus of erythrocytes, including, but not limited to, MN formation. It encompasses a range of nuclear deformities such as notched nucleus, polymorphic nucleus, binucleation, nuclear blebbing, and other morphological changes in the erythrocyte nucleus. These abnormalities serve as indicators of genotoxic or cytotoxic damage, reflecting the extent of genetic damage and chromosomal instability within the cells [9]. Genotoxicity assays, such as micronucleus testing and erythrocyte nuclear abnormality methods, are particularly valuable tools for determining cellular and genetic damage in zebrafish exposed to toxic substances [10]. Through these assays, researchers can assess the short-term and long-term genotoxic risks posed by many pesticides, such as TDML, providing valuable data that inform safety guidelines and regulatory measures. These studies are vital in shaping future agricultural practices and ensuring the safe use of chemical agents in plant protection, reducing undesirable consequences on human health and the environment.

Comparing short-term and long-term exposure effects is crucial for a comprehensive understanding of the toxicological profiles of chemicals. Short-term exposure studies focus on acute toxicity, identifying immediate effects such as oxidative stress and genotoxicity, while long-term exposure reveals the cumulative impacts, including potential chronic effects and the organism’s adaptive mechanisms [11]. For instance, *D. rerio* studies have shown that short-term exposure to certain pesticides can induce significant DNA damage, while prolonged exposure results in altered genotoxic profiles due to the activation of repair mechanisms [9,12]. In the context of fungicides like TDML, these comparisons are particularly relevant. While acute exposure may highlight direct genotoxic effects, chronic exposure studies can uncover sublethal impacts such as reproductive toxicity, morphological changes, or cumulative genetic damage [13]. Additionally, the bioaccumulation potential of TDML and its metabolic byproducts might result in effects that are only apparent after extended exposure periods [14].

Despite its widespread use, research on the genotoxic effects of TDML remains limited. In vitro studies on human lymphocyte cultures have shown that TDML can induce chromosomal aberrations, sister chromatid exchanges, and micronucleus formation, highlighting its potential as a genotoxic agent [5,15]. However, there is a lack of comprehensive data on its effects in aquatic organisms, which are particularly sensitive to environmental contamination due to their continuous exposure to waterborne pollutants. This research aims to investigate the genotoxic effects of TDML on *D. rerio* erythrocytes. In this context, the genotoxic effects of treating *D. rerio* with the determined concentrations under short-term and long-term exposures will be compared with the micronucleus (MN) test and erythrocyte nuclear anomaly (ENA) methods. In this way, the environmental risks of TDML on aquatic organisms will be better understood depending on the exposure time and data will be provided for regulatory frameworks on the safe use of agricultural chemicals.

## 2. Materials and Methods

### 2.1. Test Chemicals

**Figure 1:**
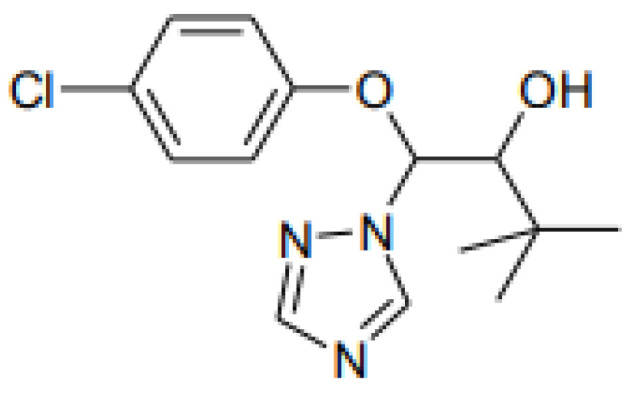
Chemical structure of TDML [16].

Triadimenol (CAS No: 55219-65-3) was used as the material in this study and purchased from a local agricultural store in Duzce, Türkiye. The chemical structure of TDML with formula C14 H18 Cl N3O2, molar mass; 295.8 g/mol is given in Figure 1. Giemsa (CAS no: 51811-82-6) and ethanol (CAS no: 64-17-5) were purchased from Merck^®^ (Darmstadt, Germany) and Sigma^®^ (St. Louis, MO, USA), respectively.

### 2.2. Concentrations and Treatment Times of Triadimenol Fungicide

The concentrations of TDML fungicide used in this study were determined as one 10th (6 mg/L), one 20th (3 mg/L) and one 40th (1.5 mg/L) based on the LC50 value of >59.15 mg/L determined for fish in previous studies [17], and the experimental groups are given in Table 1. In the present study, a total of 28 groups consisting of 1 negative control (distilled water) and 3 test groups were formed separately for each treatment period (for short-term treatment, 24, 48, 72, and 96 h; for long-term treatment, 10, 20, and 30 days). The sample size was determined according to the resource equality analysis and a total of 140 fish were used, 5 fish per group [18,19].

### 2.3. Fish Acclimation and Conditioning Process

In this study, *D. rerio* belonging to the Cyprinidae family of freshwater fish, which is the richest vertebrate family in terms of species, was used because of its fast life cycle and easy maintenance (7, 10). *D. rerio* adults (8 months old) used were obtained from a commercial certified breeding farm in Duzce (Türkiye). Before the start of the experiment, the fishes were placed in 4 different in 60 cm long × 20 cm wide × 30 cm high glass aquariums 2 weeks in advance to allow them to adapt to the environmental conditions. The aquariums were filled with settled distilled water. During the study, fish were kept in a 12:12 h photoperiod during the day and night and were fed with food (BioMar^®^, Aydin, Türkiye) once a day; water and ambient temperature were fixed at 28 ± 1 °C with thermostats and aquariums were continuously oxygenated with an air motor. The study was approved by Duzce University, Animal Experiments Local Ethics Committee (No: 2024/03/01).

### 2.4. Micronucleus (MN) Assay

For the MN assay, and blood collection, the methods of Grisolia and Cordeiro (2000) [20] and Zang et al. (2015) [21] were used with minor modifications, respectively. Accordingly, as a result of the treatments applied, blood samples (~20–25 µL) were taken from the ventral artery with a heparinized microcapillary needle, dripped onto a sterile slide, smeared, and left to dry. The dried preparations were first fixed in 98% ethanol for 10 min, then stained with 10% Giemsa stain for 20 min, passed through distilled water and allowed to dry. The preparations were examined under a light microscope (Olympus^®^ CX21, Tokyo, Japan) at ×1000 (10 × 100) magnification. A minimum of 1000 erythrocytes were assessed per fish, with abnormalities being scored in at least 200 erythrocytes from 5 randomly selected fields on each slide [22].

### 2.5. Erythrocyte Nuclear Abnormality (ENA) Assay

Blood samples taken from the experimental groups were taken from the preparations prepared for the MN test. Under the Olympus^®^ CX21 model light microscope, the erythrocyte abnormalities were examined, and abnormalities in cells and nucleus morphology were counted and photographed. To quantify the erythrocyte nuclear morphological abnormalities induced by the TDML, classification was carried out by dividing into various categories such as segmented (SN), kidney-shaped (KS), lobed (LN), blebbed (BN), notched nucleus (NN), polymorphic nucleus (PMCN), binucleated (BNC), vacuolated nucleus (VN), multinucleated cell (MNC), echinocyte (Ech), and nonnucleated (NND) and this categorization was made according to Tsarpali et al. (2020) [23] and Carrola et al. (2014) [24]. 

The electron micrographs were prepared and viewed using scanning electron microscope (FEI Quanta FEG 25, Eindhoven, The Netherland) at Duzce University, Scientific and Technologic Research Application and Research Center Laboratory. Air-dried samples were coated with gold/palladium and examined under scanning electron microscope at an accelerating voltage of 15–20 kV.

### 2.6. Statistical Analysis

Data obtained from the investigation were analyzed using one-way analysis of variance (ANOVA) in SPSS 20 for Windows (SPSS Inc., Chicago, IL, USA). They were shown as mean ± standard error (SE) and the significant difference between treated and control groups was examined by the Dunnett post hoc test. Pearson correlation analysis was used to determine the dose-response relationship. *p* < 0.05 was considered “statistically significant”.

## 3. Results

Three concentrations (1.5, 3, and 6 mg/L) of TDML fungicide were tested in *D. rerio* erythrocyte cells for short-term (24, 48, 72, and 96 h) and long-term (10, 20, and 30 days) exposure times. The data obtained regarding the total results of the study are shown in Table 2 and Table 3. TDML fungicide was tested at three concentrations (1.5, 3, and 6 mg/L) for short-term (24, 48, 72, and 96 h) and long-term (10, 20, and 30 days) exposure times on *D. rerio* erythrocyte cells. For MN assay in short-term treatments (Table 2), compared to the negative control, MN formation was found to be statistically significant (*p* ≤ 0.05) at all concentrations of TDML at 24 h. Statistical significance was determined in MN formation at the highest concentration of TDML for 48 h (*p* ≤ 0.001), at 1.5 and 3 mg/L concentrations for 72 h (*p* ≤ 0.001, *p* ≤ 0.01), and at 3 mg/L concentration for 96 h (*p* ≤ 0.05). For long-term treatments (Table 3), there were significant increases in all concentrations of TDML at 10 days (*p* ≤ 0.001), and at 1.5 and 3 mg/L concentrations for 20 days (*p* ≤ 0.001, *p* ≤ 0.05) compared to the negative control. Mortality occurred at 3 and 6 mg/L concentrations for 30 days of treatment. None of the increases in MN formation were concentration dependent except for the 48 h treatment.

According to the data obtained from the erythrocyte nucleolar abnormality assay, in short-term treatments (Table 2), the most common abnormality was SN (at 6 mg/L concentration) for 24 and 48 h followed by Ech (at 3 and 6 mg/L concentrations for 72 h; at all concentrations for 96 h). In long-term treatments (Table 3), the most common abnormality observed for 10 days of treatment was Ech at concentrations of 3 and 6 mg/L, while anucleated erythrocytes were observed at the same concentrations for 20 days of treatment. At 30 days of treatment, the most Ech abnormalities were observed at the concentration of 1.5 mg/L, while mortality occurred at all concentrations of 3 and 6 mg/L. When the first three abnormalities were ranked from most to least common for all treatment durations, the following rankings were obtained. For short-term treatments, for 24 h, SN>BNC>KS; for 48 h, SN>BNC>KS; for 72 h, Ech>NND>SN; and for 96 h, Ech>VN>SN. For long-term treatments, for 10 days, Ech>NND>VN; for 20 days, NND>Ech>VN; and for 30 days, Ech>NND>VN.

**Figure 2:**
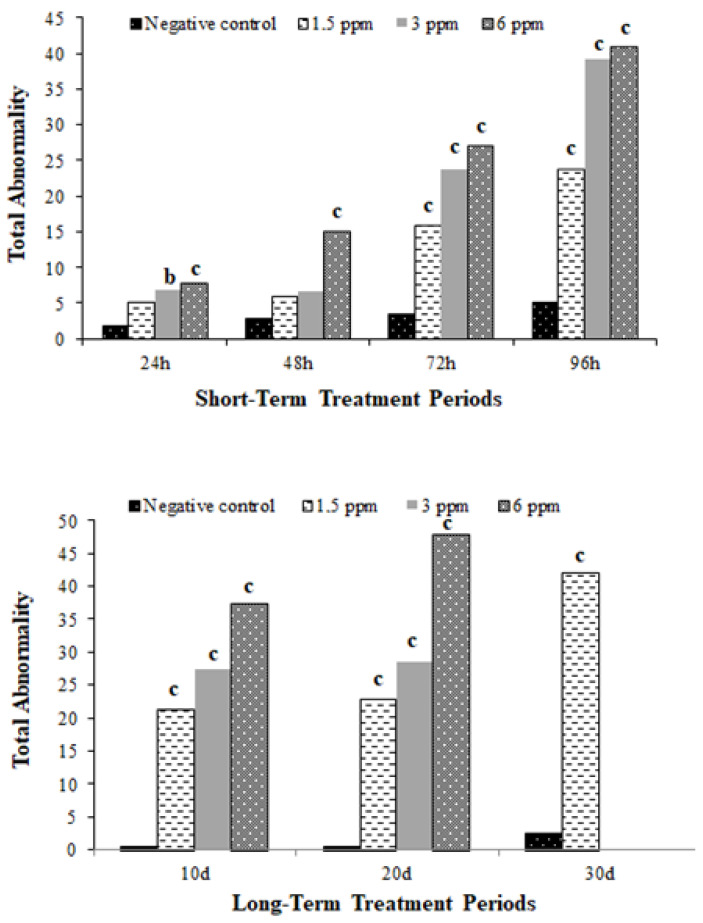
Total abnormality in *D. rerio* treated with various concentrations of TDML for short-term (24, 48, 72, and 96 h) and long-term (10, 20, and 30 days) treatment periods. b: *p* ≤ 0.01; c: *p* ≤ 0.001.

All concentrations of TDML fungicide caused an increase in the total abnormality level in *D. rerio* erythrocytes at all treatment times. These increases were concentration dependent for both short-term and long-term treatments. In short-term treatments, the increases observed at 3 and 6 mg/L concentrations for 24 h, at 6 mg/L concentrations for 48 h, and at all concentrations for 72 and 96 h were found to be statistically significant (Figure 2). 

**Figure 3:**
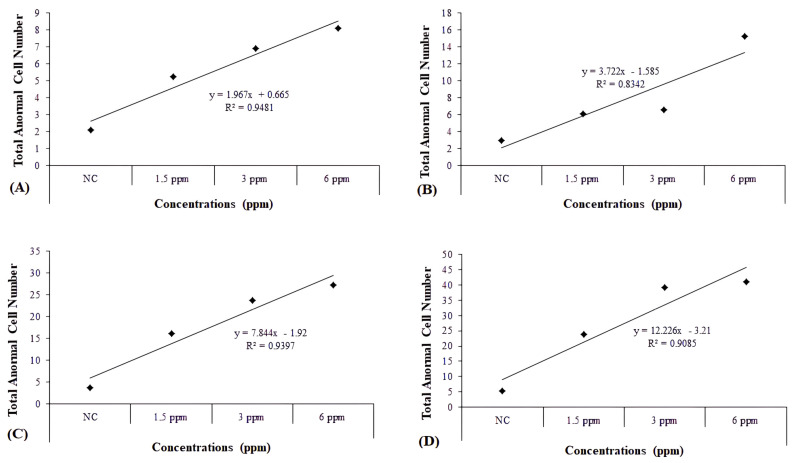
Graphs showing concentration–response curve slopes and correlation coefficients (R^2^) of total abnormality in *D. rerio* erythrocyte cells expose to TDML for 24 (**A**), 48 (**B**), 72 (**C**), and 96 h (**D**).

When the concentration–response curve slopes and correlation coefficients (R^2^) of total abnormality were evaluated, it was observed that these increases in total abnormality levels were due to increasing concentrations (R^2^ = 0.9481, *p* ≤ 0.01 for 24 h; R^2^ = 0.8342, *p* ≤ 0.01 for 48 h; R^2^ = 0.9397, *p* ≤ 0.01 for 72 h; R^2^ = 0.9085, *p* ≤ 0.01 for 96 h) (Figure 3A–D). 

**Figure 4:**
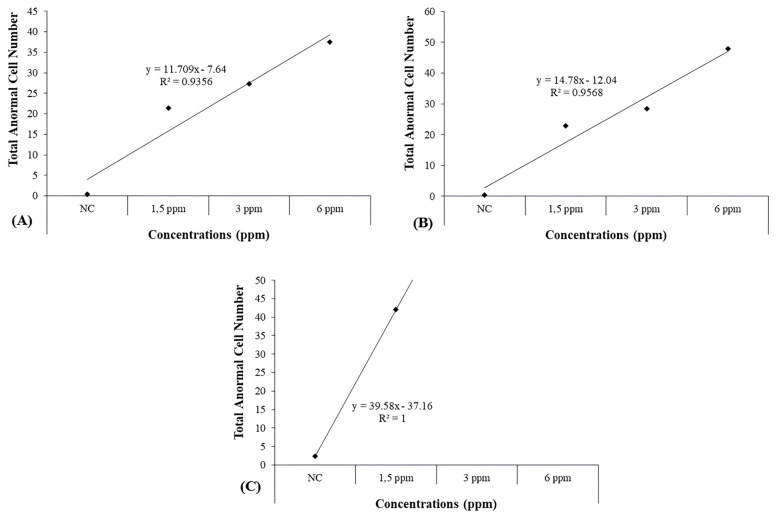
Graphs showing concentration–response curve slopes and correlation coefficients (R^2^) of total abnormality in *D. rerio* erythrocyte cells expose to TDML for 10 (**A**), 20 (**B**), and 30 d (**C**).

In long-term treatments, the increases observed at all concentrations for 10 and 20 days and at 1.5 concentration for 30 days were determined to be statistically significant Figure 2). When the concentration–response curve slopes and correlation coefficients (R^2^) of total abnormality were evaluated, it was observed that these increases in total abnormality levels were due to increasing concentrations (R^2^ = 0.9356, *p* ≤ 0.01 for 10 days; R^2^ = 0.9568, *p* ≤ 0.01 for 20 days; R^2^ = 1, *p* ≤ 0.01 for 30 days) (Figure 4A–C).

**Figure 5:**
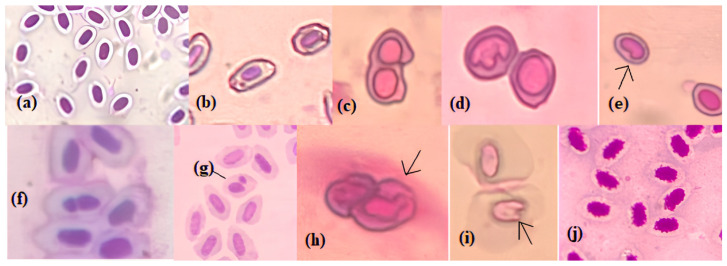
Light micrographs of erythrocyte cells of *D. rerio* (×400): (**a**) normal nucleus; (**b**) micronucleus; (**c**) binucleated cell; (**d**) polymorphic nucleus; (**e**) kidney-shaped nucleus; (**f**) segmented nucleus; (**g**,**h**) blebbed nucleus; (**i**) notched nucleus; (**j**) echinocytes.

**Figure 6:**
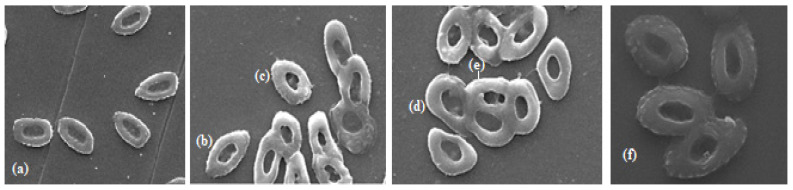
Scanning electron micrographs of erythrocyte cells of *D. rerio* (×10,000): (**a**) normal; (**b**) kidney-shaped nucleus; (**c**) notched nucleus; (**d**) polymorphic nucleus; (**e**) segmented nucleus; (**f**) echinocyte.

It is thought that the deaths that occurred at 3 and 6 mg/L concentrations for 30 days may have resulted from the increase in total abnormality levels depending on the concentration and time. Light microscope and electron microscope images of the observed ENAs are shown in Figure 5 and Figure 6, respectively.

## 4. Discussion

In this study, the genotoxic effects of various concentrations of triadimenol (TDML) on zebrafish (*Danio rerio*) erythrocytes were investigated using the micronucleus (MN) and erythrocyte nuclear abnormality (ENA) assays. Ecotoxicological research is essential for assessing the adverse impacts of environmental pollutants on ecosystems and organisms. Genotoxicity tests such as MN and ENA provide critical insights into the potential genetic damage induced by contaminants. Considering the widespread use of fungicides and their potential risks to non-target species, the results of the present study contribute to understanding the genotoxic hazards associated with TDML and underscore the need for further ecotoxicological evaluation.

The findings of this study demonstrated that TDML exposure significantly increased the frequency of MN formation in *D. rerio* erythrocytes in both short- and long-term treatments. This study is the first to assess the genotoxic effects of TDML on *D. rerio*, and there are limited studies on its genotoxicity in other non-target organisms. EPA [25] has categorized TDML as a possible human carcinogen. Similarly, tebuconazole, another triazole fungicide, has been reported to induce genotoxic and mutagenic effects in *D. rerio* when exposed to concentrations of 100, 200, and 300 µg/L for 24, 72, and 96 h [26]. Furthermore, Ascoli-Morrete et al. (2022) [4] reported that TDML increased MN formation in *Leptodactylus luctator*, while Unal et al. (2017) [5] demonstrated its clastogenic, mutagenic, aneugenic, and cytotoxic effects in cultured human lymphocytes. Additionally, Dikilitas et al. (2015) [15] and Martili (2004) [27] found dose-dependent DNA damage in mononuclear leukocytes and *Allium cepa* root tips cells, respectively, exposed to TDML.

In the present study, it was observed that MN and ENA formation was not concentration-dependent, except at 48 h. Variations in the frequency of MN and nuclear abnormalities across different concentrations and exposure durations may be attributed to the differential renewal rates of blood cells and the selective removal of damaged cells from circulation [28], which could explain the lack of a clear concentration-dependent effect.

In contrast to this research and other genotoxicity studies, some regulatory agencies [16,29] have reported that TDML is not mutagenic or genotoxic in in vitro and in vivo tests. The main reasons for these differences may be species differences in the test organisms used, exposure times, and different concentrations of TDML used. Moreover, methodological differences between in vivo and in vitro testing may also influence the results. In vitro tests are usually based on shorter exposure times and higher doses and do not take into account all biological responses of the organism. However, in vivo tests can be influenced by factors such as the organism’s immune responses, metabolic processes, and cell renewal rates.

The present study assessed the genotoxic potential of TDML using MN and NA tests in both short-term and long-term exposure periods, revealing significant genotoxic effects. The MN test is a widely used cytogenetic method to detect chromosomal damage, as micronuclei are extranuclear bodies formed from acentric chromosomal fragments or whole chromosomes that fail to segregate correctly during cell division [30]. ENAs are also considered indicators of cytotoxic and genotoxic damage in fish and serve as useful complementary endpoints to the MN test. These assays have been extensively applied in genotoxicity studies involving *D. rerio* exposed to various environmental pollutants under different experimental conditions [9,12,31,32].

Environmental pollutants such as pesticides, industrial chemicals, and food additives can induce genotoxicity by damaging DNA and proteins involved in cell division, leading to genome instability and the formation of MN and nuclear abnormalities [30]. Some pollutants contain electrophilic groups that directly bind to DNA, while non-reactive pollutants undergo metabolic activation to form reactive intermediates that damage DNA and proteins or increase ROS levels [10].

Triazole fungicides, including TDML, exert their antifungal effects by inhibiting lanosterol 14-α-demethylase (CYP51), an enzyme critical for ergosterol biosynthesis and fungal cell membrane integrity. However, triazoles have also been found to interfere with other CYP enzymes involved in steroid metabolism, sterol homeostasis, and xenobiotic detoxification. TDML disrupts ergosterol biosynthesis, leading to cell membrane instability [33].

Long-term exposure to pesticides can compromise antioxidant defense mechanisms, leading to ROS accumulation in exposed organisms. The oxidative stress generated, combined with the susceptibility of red blood cells to lipid peroxidation due to their high polyunsaturated fatty acid content, is one potential mechanism underlying the toxic effects of pesticides [28]. Therefore, TDML may induce nuclear abnormalities in zebrafish erythrocytes through mechanisms such as DNA damage, oxidative stress, cytoskeletal disruptions, and apoptosis.

The findings of this study indicate that the most frequently observed erythrocyte nuclear abnormality was Ech formation (observed at 72 and 96 h in short-term treatments and at 10 and 30 days in long-term treatments). Ech arise due to disruptions in lipid solubility within the cell membrane, ultimately leading to apoptosis [23,34], which supports the hypothesis that TDML affects cell membrane structure and function. Another prominent abnormality in this study was the presence of BNCs, which are considered the genotoxic equivalent of MN. BNCs may result from tubulin polymerization defects and impaired mitotic spindle formation, potentially caused by aneugenic toxic effects [10,28]. The presence of these abnormalities further supports the notion that TDML has the potential to induce genetic instability, which could increase the risk of carcinogenesis. These disruptions may lead to genetic instability and an increased risk of carcinogenesis [23]. This study assessed the genotoxic effects of triadimenol on zebrafish erythrocytes using micronucleus and erythrocyte nuclear abnormality assays. However, several limitations should be acknowledged. The sample size was relatively small considering the number of endpoints analyzed. A larger sample size would improve statistical power and enhance the generalizability of the findings. Mortality observed at higher concentrations after prolonged exposure was attributed to genotoxicity, yet no histopathological or biochemical validation supports this claim. The study did not measure oxidative stress markers such as superoxide dismutase, catalase, or malondialdehyde, nor were tissue histology analyses performed to confirm the cause of death. Without these assessments, the role of oxidative stress remains speculative rather than experimentally confirmed.

Another limitation concerns the environmental relevance of the tested concentrations. The study does not provide a comparison between the concentrations used in the experiments and those typically detected in aquatic environments. Future research should focus on exposure levels that more accurately reflect real-world contamination scenarios. Additionally, the physicochemical parameters of the water, such as pH, dissolved oxygen, and nitrogenous compounds, were not monitored. These factors can significantly influence both the bioavailability and toxicity of contaminants, making their omission a potential source of variability in the results.

The study was conducted on a single species and focused solely on erythrocytes, which may not fully represent the systemic effects of triadimenol in other aquatic organisms or different tissues. A broader toxicological assessment involving multiple species and additional organ systems would provide a more comprehensive understanding of its impact. Furthermore, the study was performed under controlled laboratory conditions, limiting its applicability to real-world ecological scenarios. Long-term monitoring in natural water bodies is necessary to evaluate the cumulative and interactive effects of triadimenol exposure.

The study also did not investigate the potential effects of lower, environmentally relevant doses or interactions with other contaminants. Pesticide exposure in natural ecosystems often occurs in complex mixtures rather than as a single isolated compound. Future research should explore mixture effects and lower concentration exposures to refine ecological risk assessments. Addressing these limitations will provide a more complete understanding of the genotoxic potential of triadimenol and its broader implications for aquatic health and environmental safety.

## 5. Conclusions

In conclusion, the present study provides significant evidence of the genotoxic effects of TDML fungicide on *D. rerio* erythrocytes, as demonstrated by increased MN and NA formation in both short- and long-term exposures. These findings highlight the potential risks posed by TDML to non-target aquatic organisms and reinforce the necessity of genotoxicity assessments for widely used agrochemicals. The observed nuclear abnormalities, including echinocytes and binucleated cells, suggest that mechanisms such as oxidative stress, DNA damage, and cytoskeletal disruption contribute to TDML-induced genotoxicity. Given the discrepancies in the literature regarding its genotoxic potential, further research is warranted to better understand the long-term ecological and human health implications of TDML exposure. The present study underscores the importance of the continuous monitoring and regulatory evaluation of triazole fungicides to ensure environmental and public health safety.

## Figures and Tables

**Table 1:** Experiment groups.

Group No.	Short-Term Treatment	Concentrations	Group No.	Long-Term Treatment	Concentrations
1	24 h	Negative control	17	10 days	Negative control
2		1.5 mg/L	18		1.5 mg/L
3		3 mg/L	19		3 mg/L
4		6 mg/L	20		6 mg/L
5	48 h	Negative control	21	20 days	Negative control
6		1.5 mg/L	22		1.5 mg/L
7		3 mg/L	23		3 mg/L
8		6 mg/L	24		6 mg/L
9	72 h	Negative control	25	30 days	Negative control
10		1.5 mg/L	26		1.5 mg/L
11		3 mg/L	27		3 mg/L
12		6 mg/L	28		6 mg/L
13	96 h	Negative control			
14		1.5 mg/L			
15		3 mg/L			
16		6 mg/L			

**Table 2:** The erythrocyte abnormalities induced by various concentrations of TDML in *D. rerio* for short-term (24, 48, 72, and 96 h) treatment periods.

		Micronucleated Erythrocyte		Erythrocyte Nuclear Morphological Abnormalities											TAC
TP	Conc.	MN	PMN	BNC	MNC	PMCN	SN	KS	BN	LN	NN	Ech	VN	NND	
24 h	NC	1.67 ± 0.33	0.00 ± 0.00	0.42 ± 0.15	0.00 ± 0.00	0.00 ± 0.00	0.00 ± 0.00	0.00 ± 0.00	0.00 ± 0.00	0.00 ± 0.00	0.00 ± 0.00	0.00 ± 0.00	0.00 ± 0.00	0.00 ± 0.00	2.08 ± 0.38
	1.5 mg/L	4.00 ± 0.73 ^a^	0.00 ± 0.00	0.75 ± 0.39	0.00 ± 0.00	0.00 ± 0.00	0.25 ± 0.18	0.08 ± 0.08	0.08 ± 0.08	0.08 ± 0.08	0.00 ± 0.00	0.00 ± 0.00	0.00 ± 0.00	0.00 ± 0.00	5.25 ± 0.79
	3 mg/L	4.08 ± 0.68 ^a^	0.00 ± 0.00	1.42 ± 0.47	0.08 ± 0.08	0.00 ± 0.00	0.50 ± 0.34	0.00 ± 0.00	0.67 ± 0.36 ^a^	0.00 ± 0.00	0.00 ± 0.00	0.00 ± 0.00	0.17 ± 0.17	0.00 ± 0.00	6.92 ± 0.95 ^b^
	6 mg/L	3.92 ± 0.70 ^a^	0.00 ± 0.00	0.42 ± 0.19	0.08 ± 0.08	0.00 ± 0.00	2.55 ± 1.11 ^b^	0.83 ± 0.41 ^a^	0.00 ± 0.00	0.50 ± 0.29	0.00 ± 0.00	0.00 ± 0.00	0.00 ± 0.00	0.00 ± 0.00	8.08 ± 1.77 ^c^
															
48 h	NC	1.00 ± 0.25	0.00 ± 0.00	0.67 ± 0.19	0.00 ± 0.00	0.00 ± 0.00	1.33 ± 0.22	0.00 ± 0.00	0.00 ± 0.00	0.00 ± 0.00	0.00 ± 0.00	0.00 ± 0.00	0.00 ± 0.00	0.00 ± 0.00	3.00 ± 0.44
	1.5 mg/L	2.08 ± 0.36	0.00 ± 0.00	0.83 ± 0.41	0.08 ± 0.08	0.00 ± 0.00	2.83 ± 0.58	0.25 ± 0.18	0.00 ± 0.00	0.00 ± 0.00	0.00 ± 0.00	0.00 ± 0.00	0.00 ± 0.00	0.00 ± 0.00	6.08 ± 0.93
	3 mg/L	2.91 ± 0.56	0.00 ± 0.00	0.55 ± 0.28	0.00 ± 0.00	0.09 ± 0.09	2.18 ± 0.50	0.45 ± 0.25	0.18 ± 0.12	0.18 ± 0.12	0.00 ± 0.00	0.00 ± 0.00	0.00 ± 0.00	0.00 ± 0.00	6.55 ± 0.65
	6 mg/L	7.83 ± 0.94 ^c^	0.00 ± 0.00	2.00 ± 0.89	0.08 ± 0.08	0.00 ± 0.00	4.92 ± 1.43 ^b^	0.42 ± 0.19	0.00 ± 0.00	0.00 ± 0.00	0.00 ± 0.00	0.00 ± 0.00	0.00 ± 0.00	0.00 ± 0.00	15.25 ± 2.96 ^c^
															
72 h	NC	0.58 ± 0.19	0.00 ± 0.00	0.50 ± 0.23	0.00 ± 0.00	0.00 ± 0.00	1.17 ± 0.44	0.08 ± 0.08	0.00 ± 0.00	0.42 ± 0.19	0.08 ± 0.08	0.58 ± 0.23	0.00 ± 0.00	0.25 ± 0.13	3.67 ± 0.47
	1.5 mg/L	3.91 ± 0.71 ^c^	0.00 ± 0.00	0.36 ± 0.24	0.00 ± 0.00	0.00 ± 0.00	2.45 ± 0.87	0.00 ± 0.00	0.36 ± 0.28	0.36 ± 0.24	0.00 ± 0.00	5.91 ± 2.18	0.00 ± 0.00	2.73 ± 1.32	16.09 ± 2.68 ^c^
	3 mg/L	3.64 ± 0.70 ^b^	0.00 ± 0.00	0.64 ± 0.39	0.00 ± 0.00	0.91 ± 0.91	0.55 ± 0.45	0.00 ± 0.00	1.09 ± 0.58	1.64 ± 0.74	0.00 ± 0.00	12.45 ± 2.47 ^c^	0.00 ± 0.00	2.82 ± 2.08	23.73 ± 2.39 ^c^
	6 mg/L	2.55 ± 0.73	0.00 ± 0.00	0.91 ± 0.58	0.00 ± 0.00	0.00 ± 0.00	3.64 ± 1.17	0.64 ± 0.43	0.00 ± 0.00	0.55 ± 0.39	0.09 ± 0.09	16.09 ± 2.26 ^c^	0.09 ± 0.09	2.73 ± 0.99	27.27 ± 2.90 ^c^
															
96 h	NC	0.67 ± 0.19	0.17 ± 0.11	0.50 ± 0.15	0.00 ± 0.00	0.00 ± 0.00	1.00 ± 0.25	1.00 ± 0.17	0.42 ± 0.19	0.67 ± 0.14	0.17 ± 0.11	0.50 ± 0.23	0.00 ± 0.00	0.25 ± 0.13	5.33 ± 0.48
	1.5 mg/L	1.00 ± 0.33	0.08 ± 0.08	0.42 ± 0.29	0.00 ± 0.00	0.00 ± 0.00	0.92 ± 0.29	0.91 ± 0.37	2.75 ± 0.77 ^c^	1.17 ± 0.42	0.00 ± 0.00	7.58 ± 1.45 ^c^	6.92 ± 1.76 ^c^	2.25 ± 1.39	23.92 ± 2.44 ^c^
	3 mg/L	4.33 ± 0.99 ^a^	0.00 ± 0.00	1.17 ± 0.42	0.17 ± 0.11	0.42 ± 0.29	1.33 ± 0.48	2.67 ± 0.73	0.08 ± 0.08	0.83 ± 0.41	0.00 ± 0.00	27.08 ± 1.76 ^c^	0.58 ± 0.40	0.50 ± 0.50	39.17 ± 2.10 ^c^
	6 mg/L	2.55 ± 0.47	0.00 ± 0.00	2.09 ± 0.58 ^a^	0.00 ± 0.00	0.09 ± 0.09	0.91 ± 0.37	3.09 ± 0.92	0.18 ± 0.18	0.55 ± 0.31	0.00 ± 0.00	30.91 ± 4.87 ^c^	0.64 ± 0.43	0.00 ± 0.00	41.00 ± 4.80 ^c^

TP: treatment periods; h: hour; d: day; Conc: concentrations; NC: negative control; MN: micronucleus (one); PMN: poli micronucleus (two or three); BNC: binucleated cell; MNC: multinucleated cell; PMCN: polymorphic nucleus; SN: segmented nucleus; KS: kidney-shape; BN: blebbed nucleus; LN: lobed nucleus; NN: notched nucleus; Ech: echinocyte; VN: vacuolated nucleus; NND: nonnucleated; TAC: total abnormal cell; ND: not determined due to death. (^a^) *p* ≤ 0.05; (^b^) *p* ≤ 0.01; (^c^) *p* ≤ 0.001.

**Table 3:** The erythrocyte abnormalities induced by various concentrations of TDML in *D. rerio* for long-term (10, 20, and 30 days) treatment periods.

		Micronucleated Erythrocyte		Erythrocyte Nuclear Morphological Abnormalities											TAC
TP	Conc.	MN	PMN	BNC	MNC	PMCN	SN	KS	BN	LN	NN	Ech	VN	NND	
10 d	NC	0.42 ± 0.29	0.00 ± 0.00	0.00 ± 0.00	0.00 ± 0.00	0.00 ± 0.00	0.00 ± 0.00	0.00 ± 0.00	0.00 ± 0.00	0.00 ± 0.00	0.00 ± 0.00	0.00 ± 0.00	0.00 ± 0.00	0.00 ± 0.00	0.42 ± 0.29
	1.5mg/L	6.00 ± 0.41 ^c^	1.25 ± 0.58	0.58 ± 0.23	0.00 ± 0.00	0.00 ± 0.00	1.33 ± 0.26	0.50 ± 0.15	0.25 ± 0.13	0.33 ± 0.14 ^a^	0.08 ± 0.08	2.92 ± 1.14	3.00 ± 1.44	5.08 ± 1.70	21.33 ± 2.54 ^c^
	3 mg/L	5.67 ± 0.80 ^c^	0.92 ± 0.34	1.00 ± 0.41	0.17 ± 0.11	0.00 ± 0.00	1.92 ± 0.67 ^a^	0.67 ± 0.28	0.00 ± 0.00	0.00 ± 0.00	0.17 ± 0.11	8.50 ± 1.21 ^c^	3.83 ± 0.87	4.50 ± 1.80	27.33 ± 2.99 ^c^
	6 mg/L	8.73 ± 0.85 ^c^	0.91 ± 0.41	1.00 ± 0.45	0.00 ± 0.00	0.00 ± 0.00	2.18 ± 0.71 ^b^	0.73 ± 0.24 ^a^	0.36 ± 0.20	0.09 ± 0.09	0.00 ± 0.00	9.55 ± 1.83 ^c^	7.00 ± 2.11 ^a^	6.91 ± 1.84 ^b^	37.45 ± 3.43 ^c^
															
20 d	NC	0.33 ± 0.26	0.00 ± 0.00	0.00 ± 0.00	0.00 ± 0.00	0.00 ± 0.00	0.00 ± 0.00	0.00 ± 0.00	0.00 ± 0.00	0.00 ± 0.00	0.08 ± 0.08	0.00 ± 0.00	0.00 ± 0.00	0.00 ± 0.00	0.42 ± 0.26
	1.5 mg/L	5.00 ± 0.71 ^c^	1.56 ± 0.38 ^c^	1.30 ± 0.37 ^c^	0.00 ± 0.00	0.60 ± 0.40	2.20 ± 0.36 ^c^	1.10 ± 0.28 ^b^	1.60 ± 0.96	0.50 ± 0.27	0.20 ± 0.13	1.70 ± 1.70	6.80 ± 3.33	0.50 ± 0.50	22.90 ± 4.03 ^c^
	3 mg/L	2.50 ± 0.56 ^a^	0.67 ± 0.28	0.25 ± 0.18	0.00 ± 0.00	0.08 ± 0.08	1.25 ± 0.49 ^a^	0.67 ± 0.22	1.50 ± 0.50	0.83 ± 0.34	0.42 ± 0.34	6.92 ± 2.21 ^a^	1.08 ± 0.56	12.33 ± 2.89 ^b^	28.50 ± 2.77 ^c^
	6 mg/L	2.00 ± 0.57	0.36 ± 0.15	0.27 ± 0.19	0.00 ± 0.00	3.91 ± 1.18 ^c^	2.09 ± 0.41 ^c^	0.73 ± 0.30	1.27 ± 0.66	0.82 ± 0.33	0.09 ± 0.09	6.27 ± 2.45	5.64 ± 2.57	24.36 ± 4.29 ^c^	47.82 ± 3.87 ^c^
															
30 d	NC	0.00 ± 0.00	0.00 ± 0.00	0.42 ± 0.15	0.00 ± 0.00	0.17 ± 0.11	0.33 ± 0.14	0.50 ± 0.15	0.25 ± 0.13	0.33 ± 0.14	0.00 ± 0.00	0.00 ± 0.00	0.17 ± 0.11	0.25 ± 0.13	2.42 ± 0.34
	1.5 mg/L	1.33 ± 0.49	0.00 ± 0.00	0.17 ± 0.17	0.00 ± 0.00	0.67 ± 0.67	2.67 ± 1.02	0.00 ± 0.00	0.33 ± 0.14	0.00 ± 0.00	0.00 ± 0.00	21.33 ± 1.89 ^c^	4.33 ± 1.48	11.17 ± 2.59 ^c^	42.00 ± 3.46 ^c^
	3 mg/L	ND	ND	ND	ND	ND	ND	ND	ND	ND	ND	ND	ND	ND	ND
	6 mg/L	ND	ND	ND	ND	ND	ND	ND	ND	ND	ND	ND	ND	ND	ND

TP: treatment periods; h: hour; d: day; Conc: concentrations; NC: negative control; MN: micronucleus (one); PMN: poli micronucleus (two or three); BNC: binucleated cell; MNC: multinucleated cell; PMCN: polymorphic nucleus; SN: segmented nucleus; KS: kidney-shape; BN: blebbed nucleus; LN: lobed nucleus; NN: notched nucleus; Ech: echinocyte; VN: vacuolated nucleus; NND: nonnucleated; TAC: total abnormal cell; ND: not determined due to death. (^a^) *p* ≤ 0.05; (^b^) *p* ≤ 0.01; (^c^) *p* ≤ 0.001.

## Data Availability

The original contributions presented in this study are included in the article. Further inquiries can be directed to the corresponding author.
